# Administration of Anti–SARS-CoV-2 Monoclonal Antibodies After US Food and Drug Administration Deauthorization

**DOI:** 10.1001/jamanetworkopen.2022.28997

**Published:** 2022-08-29

**Authors:** Timothy S. Anderson, Ashley O’Donoghue, Oren Mechanic, Tenzin Dechen, Jennifer Stevens

**Affiliations:** 1 Division of General Medicine, Beth Israel Deaconess Medical Center, Boston, Massachusetts; 2 Center for Healthcare Delivery Science, Beth Israel Deaconess Medical Center, Boston, Massachusetts; 3 Harvard Medical Faculty Physicians at Beth Israel Deaconess Medical Center, Boston, Massachusetts; 4 Department of Emergency Medicine, Mount Sinai Medical Center, Miami Beach, Florida; 5 Division of Pulmonary, Critical Care and Sleep Medicine, Beth Israel Deaconess Medical Center, Boston, Massachusetts

## Abstract

This cross-sectional study uses time-series data to evaluate the administration of bamlanivimab-etesevimab and casirivimab-imdevimab monoclonal antibody treatments for SARS-CoV-2 infection after the US Food and Drug Administration deauthorized their use in early 2022.

## Introduction

In 2021, the US Food and Drug Administration (FDA) issued emergency use authorizations for 2 anti–SARS-CoV-2 monoclonal antibodies (mAbs), bamlanivimab-etesevimab and casirivimab-imdevimab, to treat mild to moderate COVID-19 in high-risk ambulatory patients. The Omicron variant was determined to not be susceptible to these treatments, leading the FDA to deauthorize their use on January 24, 2022.^1^ Given public controversy over this decision,^2^ we evaluated the administration of bamlanivimab-etesevimab and casirivimab-imdevimab in the US after FDA deauthorization.

## Methods

We conducted a serial cross-sectional study using the US Department of Health and Human Services COVID-19 Reported Patient Impact and Hospital Capacity by State Timeseries data set.^3^ All US hospitals and health systems are required to report the number of therapeutic courses of bamlanivimab-etesevimab and casirivimab-imdevimab administered in the prior week and the number currently on hand. Because these data are publicly available and aggregated, this study was exempt from institutional review board approval and informed consent was waived in accordance with the Common Rule. The study followed the STROBE reporting guideline.

We examined reporting from October 27, 2021, through June 29, 2022, to identify national trends in weekly use of each mAb product before and after FDA deauthorization. We report the total doses administered and the ratio of mAbs used to COVID-19 cases reported.^4^ For each state, we identified the number of mAb doses administered after deauthorization and the proportion of mAb doses reported as on hand that were administered during this period. Calculations were performed with Microsoft Excel.

## Results

**Figure 1.**
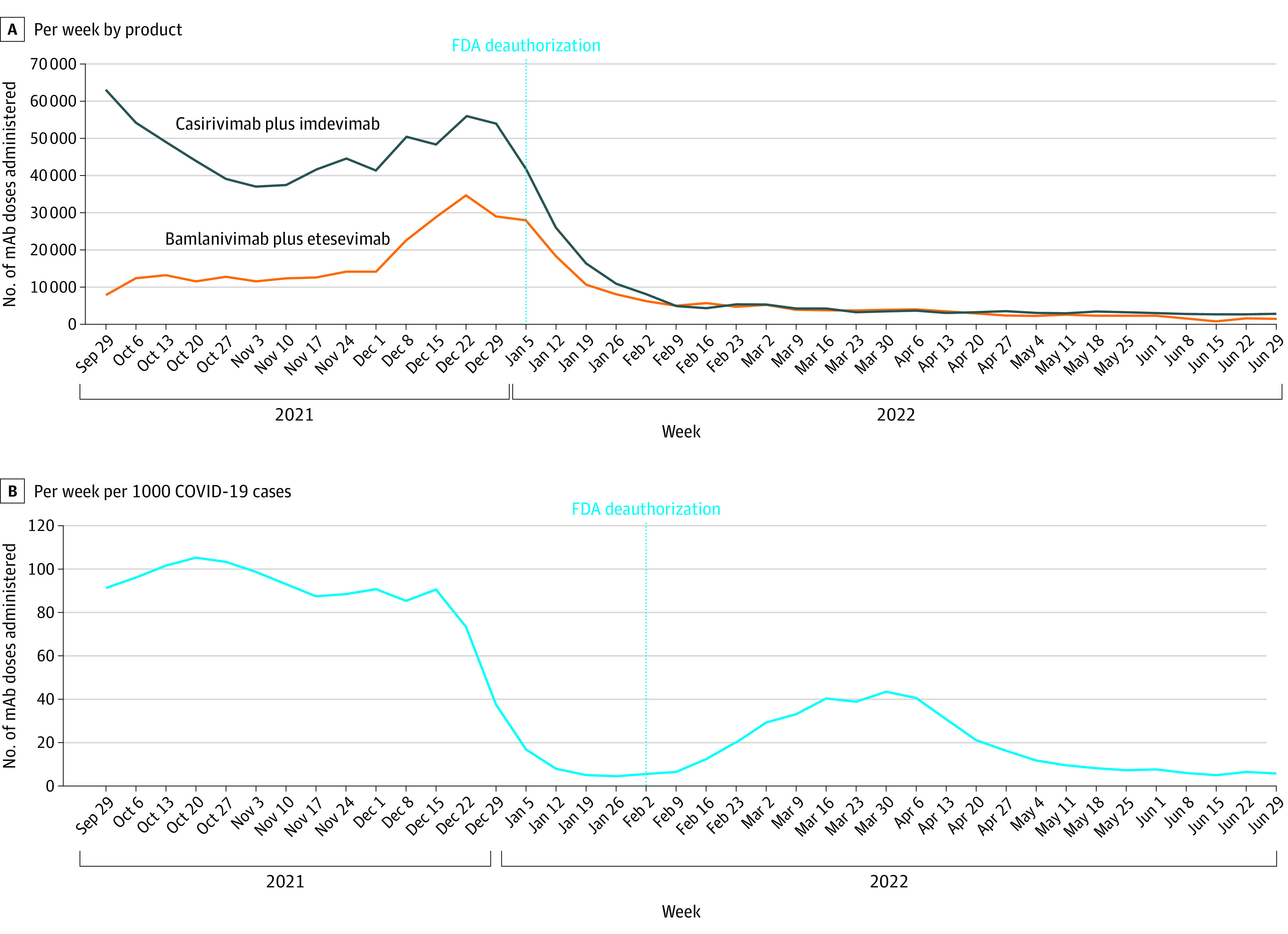
Weekly Administration of Monoclonal Antibody (mAb) Doses, by Hospitals and Health Systems A, Number administered, by mAb product. B, Number administered per 1000 COVID-19 cases. FDA indicates US Food and Drug Administration.

Administration of bamlanivimab-etesevimab and casirivimab-imdevimab peaked during the week of December 22, 2021, with 91 036 total doses reported. Use of mAbs declined throughout the remainder of the study period. Both bamlanivimab-etesevimab and casirivimab-imdevimab continued to be administered after FDA deauthorization on January 24, 2022 (Figure 1A). Overall use of these mAbs gradually declined after FDA deauthorization, whereas the proportion of COVID-19 cases for which these deauthorized treatments were used peaked in late March 2022, at 43 treatments per 1000 cases (Figure 1B).

**Figure 2.**
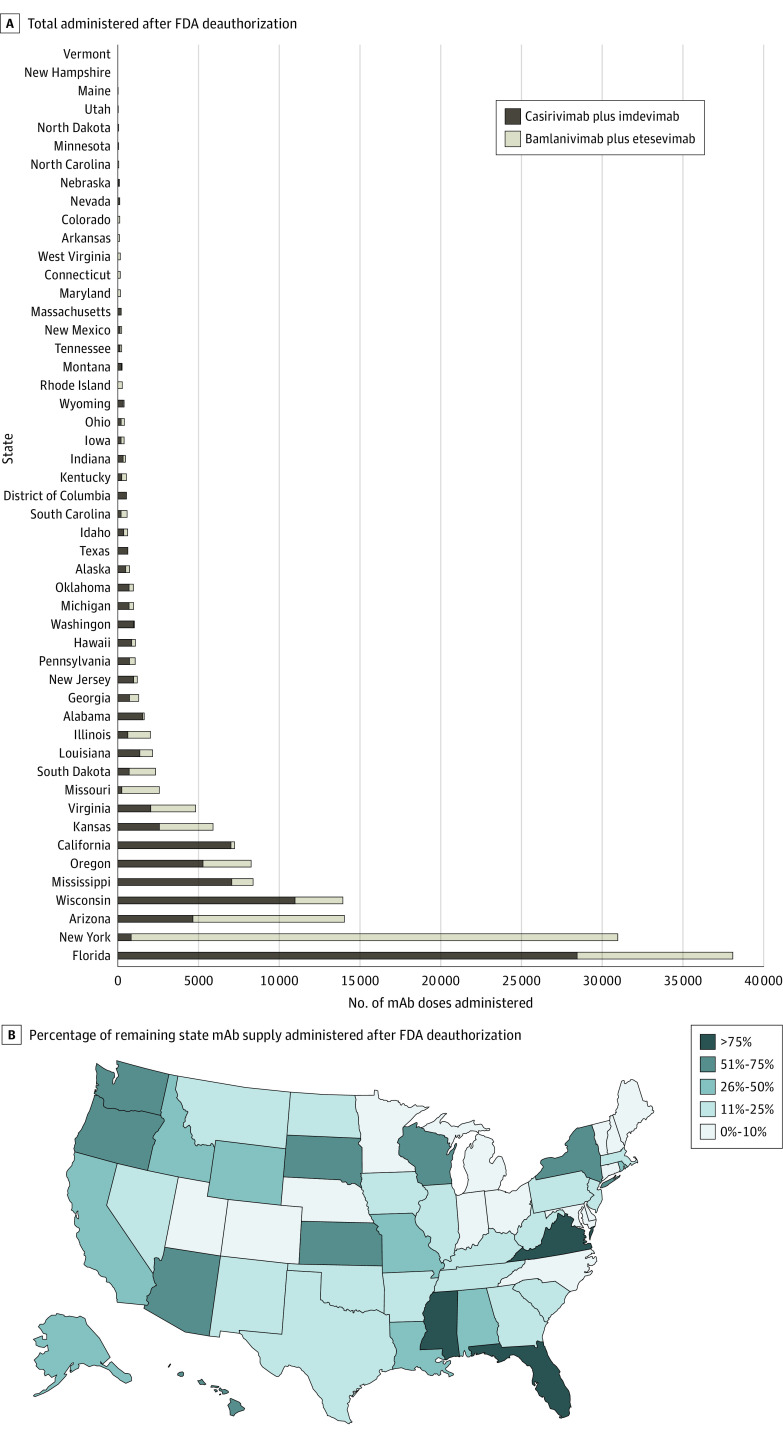
Monoclonal Antibody (mAb) Doses Administered After US Food and Drug Administration (FDA) Deauthorization, by State A, Total number administered. B, Percentage of mAb supply administered.

A total of 158 395 mAb doses were administered after deauthorization. There was wide variability in mAb administration by state, with Florida and New York accounting for 24% and 20%, respectively (Figure 2A). Fourteen states administered less than 10% of their remaining supply, and 11 states administered more than 50% of their remaining supply (Figure 2B).

## Discussion

According to the results of this serial cross-sectional study, hospitals and health systems administered more than 158 000 anti–SARS-CoV-2 mAb doses in early 2022, despite FDA deauthorization because of a lack of efficacy against the Omicron variant. Medicare payments for mAb administration range from $450 to $750 per dose,^5^ indicating that spending on these deauthorized treatments likely exceeds $71 million. Our findings suggest that the use of deauthorized mAb products was widespread, even though patients had a minimal likelihood of benefit. Whether deauthorized treatments will be covered by payers and whether the FDA will take regulatory action against entities violating its guidance remains unknown.

The continued use of deauthorized anti–SARS-CoV-2 mAb treatments may reflect conflicting state government guidance,^2^ lack of hospital awareness of deauthorization, or other factors. Although the FDA announcements clearly stated that these mAbs were no longer authorized for use, the agency did not fully revoke their emergency use authorizations because of the possibility that future COVID-19 variants could retain susceptibility, which could have led to misinterpretation.^1^

Limitations of this study include a reliance on hospital reporting, which does not include other mAb administration sites (eg, federal health systems and correctional facilities). Reporting data are not yet publicly available for other COVID-19 therapeutics. All public data on mAb administration are aggregated to the state level; thus, we were unable to explore facility variation in mAb use. Efforts to improve transparency, equity, and value in the COVID-19 response should include public release of facility-level reporting for all therapeutics.
